# Resident Microbiome Disruption with Antibiotics Enhances Virulence of a Colonizing Pathogen

**DOI:** 10.1038/s41598-017-16393-3

**Published:** 2017-11-23

**Authors:** Courtney A. Thomason, Nathan Mullen, Lisa K. Belden, Meghan May, Dana M. Hawley

**Affiliations:** 1 Department of Biological Sciences, Virginia Tech, Blacksburg, VA USA; 2 Department of Biomedical Sciences, University of New England, Biddeford, ME USA

## Abstract

There is growing evidence that symbiotic microbes play key roles in host defense, but less is known about how symbiotic microbes mediate pathogen-induced damage to hosts. Here, we use a natural wildlife disease system, house finches and the conjunctival bacterial pathogen *Mycoplasma gallisepticum* (MG), to experimentally examine the impact of the ocular microbiome on host damage and pathogen virulence factors during infection. We disrupted the ocular bacterial community of healthy finches using an antibiotic that MG is intrinsically resistant to, then inoculated antibiotic- and sham-treated birds with MG. House finches with antibiotic-disrupted ocular microbiomes had more severe MG-induced conjunctival inflammation than birds with unaltered microbiomes, even after accounting for differences in conjunctival MG load. Furthermore, MG cultures from finches with disrupted microbiomes had increased sialidase enzyme and cytadherence activity, traits associated with enhanced virulence in *Mycoplasmas*, relative to isolates from sham-treated birds. Variation in sialidase activity and cytadherence among isolates was tightly linked with degree of tissue inflammation in hosts, supporting the consideration of these traits as virulence factors in this system. Overall, our results suggest that microbial dysbiosis can result in enhanced virulence of colonizing pathogens, with critical implications for the health of wildlife, domestic animals, and humans.

## Introduction

Animals harbor diverse symbiotic microbes that serve key roles in host defense against pathogens^1^. Commensal microbes promote innate and adaptive immune responses in vertebrates^2,3^, and intact microbiomes can minimize pathogen invasion via colonization resistance^4,5^. However, little is known about the role of symbiotic microbes in mediating pathogen virulence or pathogenicity following successful host invasion. Understanding how microbiomes influence the level of harm that pathogens cause their hosts is critical to fully elucidating the role of microbial symbionts in host health.

Virulence, defined here as the level of damage that pathogens cause their hosts, is a function of intrinsically-linked host and pathogen traits^6^. Symbiotic microbes can influence pathogen virulence via several non-mutually exclusive pathways. First, symbionts can limit pathogen growth^7^ via diverse mechanisms including interbacterial warfare, metabolic defense, or physical interference^8,9^. These effects minimize a host’s pathogen burden, which often underlies the extent of host exploitation and virulence^10,11^. Symbiotic microbes can also alter host immune responses^12,13^, in some cases dampening host inflammation in both autoimmune and infectious disease^14–17^. Thus, for pathogens where host damage is largely a result of overactive immune responses^6^, symbiotic microbes may minimize virulence via immune-mediated effects. Finally, symbiotic microbes could directly or indirectly alter the expression or activity of pathogen virulence factors. For example, endophytic bacteria of plants can directly quench the quorum sensing molecules of an invading bacterial species^18^. Similarly, virulence of the invasive enteric pathogen *Salmonella enterica* is reduced in the presence of a small molecule produced by commensal *Clostridium* species in the human gut, which minimizes host cell invasion and expression of genes linked with pathogenicity^19^. In other cases, effects of the microbiome on pathogen virulence might be indirect. For example, the intestinal opportunistic pathogen *Clostridium difficile* regulates toxin production via quorum-sensing mechanisms^20^, which are likely to be enhanced following disruption of intestinal microbial symbionts. However, the role of microbiome disruption in modulating *C. difficile* toxin production has not been directly addressed.

Here, we address the role of conjunctival bacterial symbionts in mediating virulence in a naturally occurring, yet experimentally tractable, wildlife disease system: house finches and the conjunctival bacterial pathogen *Mycoplasma gallisepticum* (MG). MG has caused annual epidemics of conjunctivitis in free-living finches since the mid-1990s^21^, reducing finch over-winter survival^22^ and causing significant host population declines^23^. Mycoplasmal conjunctivitis is a highly inflammatory disease, producing severe conjunctival swelling, local heterophilic and lymphophilic infiltrates^24^, and systemic pro-inflammatory cytokine expression^25^. Thus, overreactive host immune responses appear to contribute significantly to host damage, or virulence, in this system. From the pathogen side, key virulence factors of *Mycoplasmas* can be characterized phenotypically^26,27^, and include cytadherence (ability to bind to host cells^28^) and sialidase activity (associated with pathogen colonization, nutrition, and cellular degradation^29^). Because colonization and pathogenesis traits of MG vary rapidly *in vivo* during infection in poultry^30^, we predicted that the activity of MG virulence factors during infection might be influenced (either directly or indirectly via changes in tissue architecture) by bacterial symbiont dysbiosis.

We previously used 16S rRNA gene amplicon sequencing to characterize the conjunctival microbiome of captive house finches both prior to and following experimental inoculation with MG^31^. Similar to other vertebrate ocular microbiomes, the bacterial ocular microbiome in healthy house finches was dominated by taxa in the phyla Firmicutes and Proteobacteria^32–34^. In healthy finches, the genus *Lactococcus* made up the vast majority of relative abundance (~75%)^31^. Furthermore, experimental infection with MG resulted in detectable changes in relative abundance of the resident microbiota, indicating the potential for interactions between MG and resident symbiotic bacteria. However, experimental manipulations of resident symbionts are critical to determine whether ocular symbionts play a causative role in mediating the outcome of MG infection.

**Figure 1:**
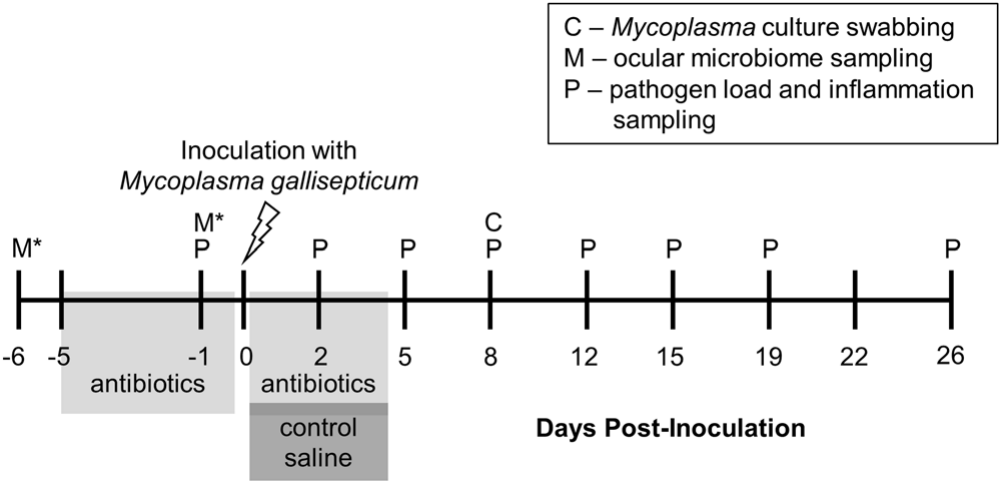
Timeline for sampling house finches throughout an experimental mycoplasmal conjunctivitis infection. *Indicates ocular microbiome sampling for a separate set of birds to determine the sensitivity of the ocular microbiome to β-lactam antibiotic treatment (see Supplement).

In the present study, we asked whether the ocular bacterial microbiome of house finches mediates the degree of host damage and the activity of pathogen virulence factors during experimental MG infection. We disrupted the conjunctival bacterial microbiome of healthy house finches using local treatment with the broad-spectrum antibiotic cefazolin, which is a β-lactam antibiotic that MG is not sensitive to (see *Methods*). We confirmed the sensitivity of members of the finch ocular microbiome to this antibiotic using culture-based methods *in vitro* and *in vivo* (see Supplementary Material). We used two lengths of antibiotic treatment to account for the possibility that resident ocular bacteria might recover quickly from perturbation: antibiotics for only the five days prior to MG inoculation (*Short Antibiotics*) or antibiotics for five days prior to and four days following MG inoculation (*Long Antibiotics;* see *Methods*). In a full factorial design (Table 1), we inoculated individuals harboring either intact or perturbed microbiomes with MG or media alone and assessed the inflammation severity of host tissue (quantified as “conjunctival inflammation scores”) and conjunctival pathogen burden throughout the course of infection (Fig. 1). We also isolated MG from the conjunctiva of all MG-treated birds on day 8 to quantify phenotypic activity of two virulence factors of *Mycoplasmas* (sialidase enzyme and cytadherence activity).

**Table 1:** Experimental groups testing the effect of antibiotic-disrupted resident microbiomes on *Mycoplasma gallisepticum* (MG) virulence.

	Sham	MG
*No Antibiotics* (Control)	N = 10 (6:4)	N = 10 (6:4)
*Short Antibiotics* (Pre-MG only)	N = 10 (5:5)	N = 10 (5:5)
*Long Antibiotics* (Pre + Post-MG)	N = 10 (5:5)	N = 10 (5:5)

Sex ratios listed as (male:female). All birds in the “MG” treatments were confirmed naïve to the pathogen at capture (see Supplement).

## Results

### Antibiotic Treatment and Development of Pathology

**Figure 2:**
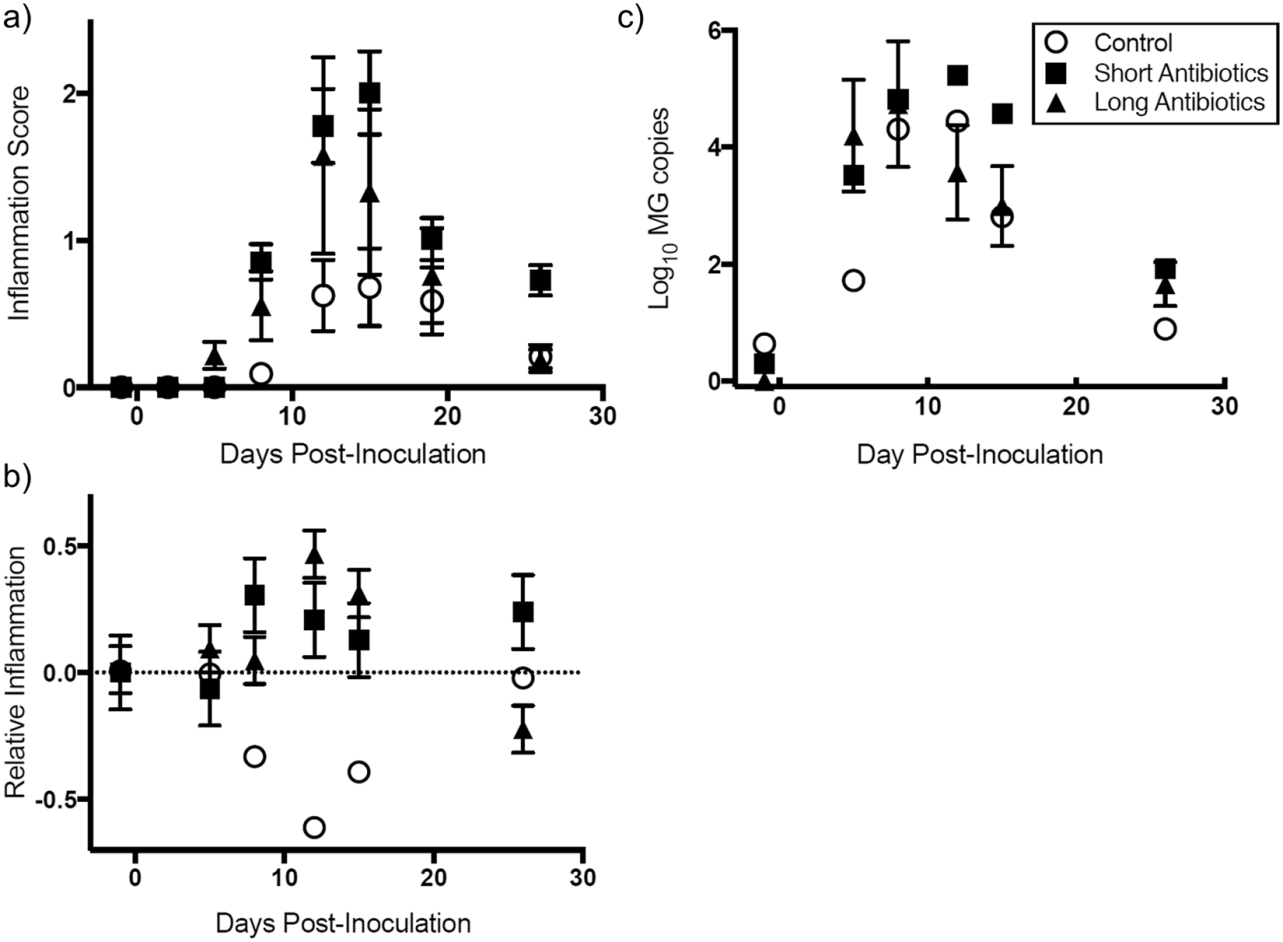
Effects (LSmeans ± standard error) of β-lactam antibiotic perturbation on development of mycoplasmal conjunctivitis in house finches inoculated with *Mycoplasma gallisepticum* (MG). (**a**) Inflammation severity is significantly higher for finches that received antibiotic treatment. (**b**) Relative inflammation, which controls for differences in pathogen load, is also significantly more severe for finches that received antibiotics to perturb their resident ocular microbiome. (**c**) Finally, pathogen load increases significantly earlier in MG infection in finches that received antibiotic treatment. Note: some error bars are too small to see. Sham-inoculated birds (Table 1) are not shown.

Finches that received topical cefazolin antibiotics to disrupt the resident ocular microbiome developed higher conjunctival inflammation scores following MG inoculation than control finches that did not receive topical antibiotics, and this effect varied with time (antibiotics × PID: Χ^2^ = 30.46, df = 14, *p* = 0.006; Fig. 2a). Post-hoc contrasts indicated that the effect of antibiotics on inflammation was strongest on days 12 and 15 post-inoculation, and largest for the *Short Antibiotics* treatment (Supplementary Table S1). However, no statistical differences were detected in inflammation scores between the two antibiotic treatment groups (*Short* versus *Long*: *p* > 0.13). No conjunctival inflammation was ever detected in finches that received sham MG inoculations, regardless of antibiotic treatment, indicating that the antibiotic treatment itself did not induce quantifiable localized inflammation.

Relative inflammation severity, a metric of inflammation score which controls for conjunctival pathogen burden (see *Methods*), was also higher in antibiotic-treated birds relative to controls, and this pattern also varied with time (antibiotics × PID: Χ^2^ = 20.46, df = 10, *p* = 0.025; Fig. 2b). Post-hoc contrasts indicated that the effects of antibiotic treatment on relative inflammation severity were strongest at day 12 post-infection (Supplementary Table S2).

Antibiotic-treated birds had significantly higher pathogen loads compared to control birds following inoculation with equal doses of MG, but this effect varied with time (antibiotics × PID: Χ^2^ = 30.4, df = 10, *p* < 0.001; Fig. 2c). Post-hoc tests indicated that the effects of antibiotics on pathogen load were strongest at day 5 post-infection (Supplementary Table S2); by day 8, pathogen loads were similar among all three treatments.

### MG virulence factors

**Figure 3:**
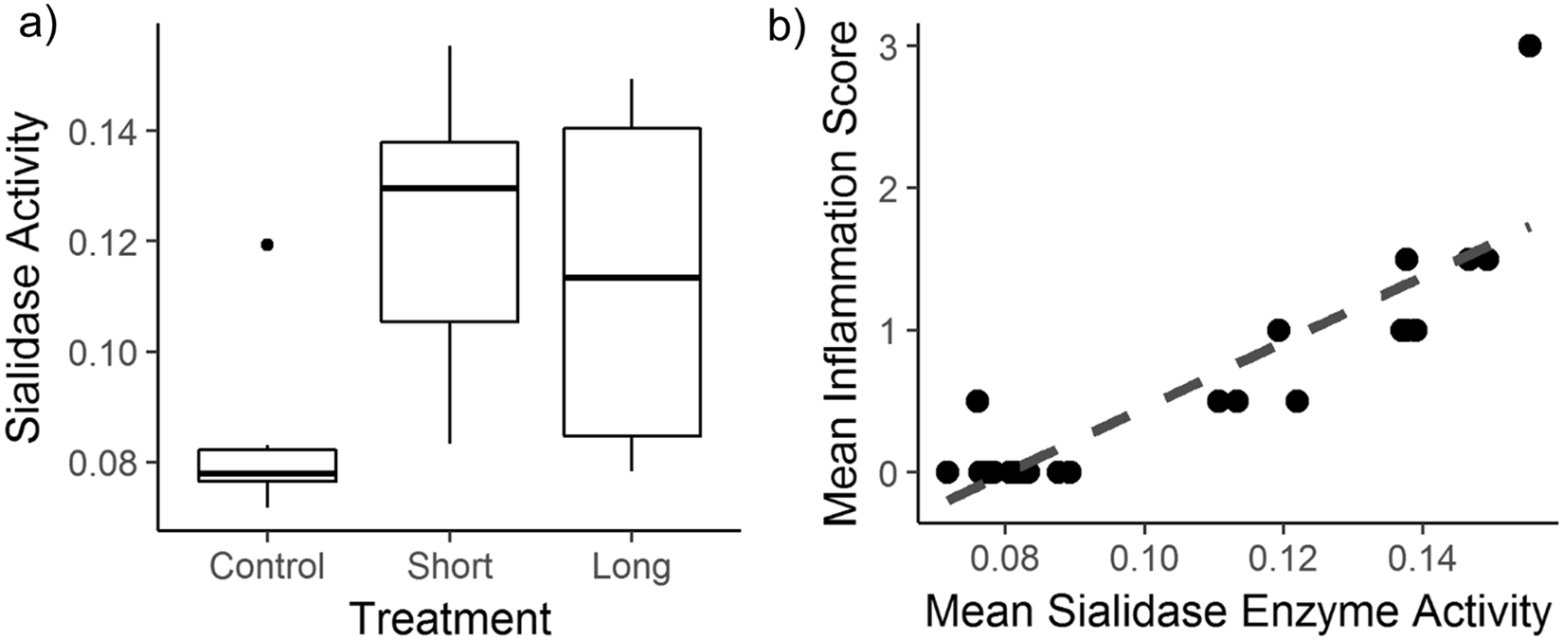
Effects of prior β-lactam antibiotic perturbation on *Mycoplasma gallisepticum* sialidase activity, and the relationship between sialidase activity and tissue inflammation at the time of MG isolation. (**a**) *Mycoplasma gallisepticum* recovered from house finches treated with topical antibiotics (*Short* + *L* *ong* treatments) to disrupt their resident ocular microbiomes had enhanced sialidase enzyme activity (mean ± standard error) on day 8 post-inoculation. (**b**) Sialidase activity of recovered isolates positively correlated with host tissue inflammation on the day of isolation. Because sialidase and cytadherence were strongly correlated (R = 0.98) and thus cannot be considered as independent, results are shown for sialidase enzyme activity only.

MG isolated from conjunctivae of birds previously treated with cefazolin antibiotics had higher sialidase enzyme activity (Χ^2^ = 13.7, df = 2, *p* = 0.001; *Short*: *p* = 0.001, *Long*: *p* = 0.02; Fig. 3a) and cytadherence activity (Χ^2^ = 12.4, df = 2, *p* < 0.001; *Short*: *p* = 0.003, *Long*: *p* = 0.02). Sialidase enzyme activity and cytadherence were highly correlated with each other (Pearson correlation: 0.976, t = 21.8, df = 23, *p* < 0.001). Interestingly, host variation in inflammation score on the day of MG isolation (day 8) correlated strongly with both sialidase enzyme activity (F = 86.9, df = 1, *p* < 0.001, R^2^ = 0.79, Fig. 3b) and cytadherence (F = 77.1, df = 1, *p* < 0.001, R^2^ = 0.76), which suggests that the phenotypic changes of MG recovered from antibiotic-treated finches may be linked to the increased inflammation severity also found in antibiotic-treated birds. No significant differences were detected in sialidase enzyme activity or cytadherence between the two antibiotic treatment groups (*Short* versus *Long*: *p* > 0.76).

## Discussion

Antibiotic-induced dysbiosis of the resident ocular microbiome in house finches resulted in more severe infection-induced tissue damage and enhanced activity of pathogen virulence factors during experimental infection with a commonly occurring ocular bacterial pathogen, MG. House finches treated with a broad-spectrum β-lactam antibiotic, cefazolin, that MG is intrinsically resistant to, showed significantly more severe conjunctival inflammation, even after accounting for differences in pathogen burden. Furthermore, MG isolated from antibiotic-treated house finches showed increased activity of two important virulence factors of *Mycoplasmas* – sialidase enzyme activity and cytadherence – and these phenotypic traits correlated strongly and positively with host variation in tissue inflammation severity. Together, these results suggest that intact ocular microbiomes mitigate the development of virulent Mycoplasmal conjunctivitis in house finches.

Our results indicate that the ocular microbiome influences two components of host response that we know are critical to virulence in this system. First, we found that MG burden in the conjunctivae, early in infection, was higher for antibiotic-treated birds. These results indicate that a perturbed ocular microbiome facilitates higher host exploitation, or pathogen growth, which is strongly correlated with virulence in this system^35^. Second, we found that antibiotic perturbation increased the extent of conjunctival inflammation during MG infection. Because pathogen load and inflammation scores are tightly linked in this system^35^, we also quantified relative inflammation severity as a metric of the degree of inflammation present at a given pathogen burden. Interestingly, relative inflammation severity was also significantly higher in cefazolin-treated birds, suggesting that microbiome disruption has direct effects on conjunctival inflammation that may be independent of changes in pathogen burden. Because conjunctivitis severity predicts ease of capture during a mock-predation event^36^, the degree of conjunctival inflammation is a robust proxy for the fitness impacts of MG infection in the wild (the ecological definition of virulence^37^). Thus, our results suggest that an intact ocular microbiome not only minimizes tissue damage, but also the negative fitness impacts of MG infection in free-living populations. Overall, our results, combined with recent evidence that the ocular microbiome is protective against *Pseudomonas aeruginosa*-induced keratitis in mice^17^, suggest that the ocular microbiome serves a key protective function similar to that documented for several other mucosal microbiomes^14,19,20^.

Antibiotic treatment also enhanced the activity of two *Mycoplasma* virulence factors, sialidase activity and cytadherence. These phenotypic changes in MG are not likely to be a direct result of antibiotic treatment because MG is intrinsically resistant to this particular antibiotic, which was confirmed by our pathogen load results (Fig. 2c). Furthermore, the detected phenotypic changes were equivalent for both lengths of antibiotic treatment, indicating that phenotypic changes occurred even when MG itself was never directly exposed to antibiotics (e.g., *Short Antibiotics*, given pre-MG only). Because we had to propagate the pathogen prior to assessing virulence phenotypes, our results should be interpreted with some caution. However, if passage alone produced the detected changes in sialidase activity, those changes would have occurred in our control group as well. Additionally, Pflaum *et al*.^30^ found that one *in vitro* passage of MG did not immediately cause reversion to MG’s axenic culture gene expression profile, suggesting that propagating MG prior to assessing sialidase and cytadherence activity is unlikely to have significantly impacted our results. Sialidase enzyme activity and cytadherence are broadly associated with pathogen virulence, and specifically appear to be important virulence factors for *Mycoplasmas* ^38,39^. For example, MG sialidase gene knockouts cause significantly less severe disease in chickens^27^. Similarly, transforming an avirulent strain of poultry MG with the wild-type cytadhesin *gapA* operon restored cytadherence ability and virulence to a level comparable to the wild-type MG strain^26^. Intriguingly, variation in sialidase activity and cytadherence amongst isolates correlated strongly with individual variation in host disease response at the same time point. Although prior studies of sialidase activity have used poultry-lineage MG, our results suggest that sialidase activity is also associated with virulence in house finch-clade MG, a monophyletic clade derived from poultry clade-MG in the mid-1990s^40^. These results suggest that microbial dysbiosis, either via direct or indirect mechanisms (i.e., changes in tissue architecture), can result in enhanced virulence of a colonizing pathogen.

Further work is needed to determine both the symbiotic taxa and specific mechanisms responsible for the observed effects of the microbiome on pathogen load and virulence. Our 16S rRNA gene amplicon data indicated that the majority of the relative abundance in the ocular communities at the start of our experiment (~76%) was *Lactococcus* spp., which is similar to our prior findings^31^. *Lactococcus* spp. are cultivable lactic acid bacteria that we successfully cultured from finch eye swabs, and pure cultures of house finch *Lactococcus* spp. were found to be highly sensitive to cefazolin (MIC = 0.5 μg/mL; see Supplement). Although we were unable to quantify the extent to which specific taxa were reduced in abundance or viability by antibiotic treatment, the strong community dominance of *Lactococcus* spp., combined with its high sensitivity to cefazolin, together suggest that the reduced viability of the house finch ocular microbiome following antibiotic treatment (see Supplement) was likely driven by responses of *Lactococcus* spp. in particular. However, taxon-specific assays are needed to validate this claim. *Lactococcus* species, like other lactic acid producing bacteria, produce antimicrobial metabolites that can inhibit growth of some pathogenic bacteria, and are beneficial members of animal microbiomes^41,42^. Resident *Lactococcus* in the house finch ocular microbiome may play a similar role, but taxon-specific augmentation studies are needed to determine whether *Lactococcus* or other symbionts have key roles in the detected changes in disease severity during MG infection.

Overall, our results suggest that ocular microbial dysbiosis enhances the severity of Mycoplasmal conjunctivitis and the expression of pathogen virulence factors. In this system, more severe conjunctival inflammation facilitates pathogen deposition by finches onto environmental fomites^43^. Thus, the effects of microbial dysbiosis on inflammation severity could have important downstream impacts on both the fitness effects of MG infection in free-living birds, and the likelihood of ongoing transmission. A growing list of studies have found intact microbiomes play a protective role against pathogens, including egg microbiomes of endangered sea turtles that protect against the emerging pathogen *Fusarium falciforme* ^44^, golden frog skin microbiomes that protect against chytrid fungus^45^, and the human sinus microbiome that protects against bacteria causing chronic sinusitis^46^. Our results suggest that antibiotic-induced bacterial dysbiosis can have a suite of important effects on host and pathogen responses during the infection process, with far-reaching consequences for host fitness and disease dynamics. As infectious diseases continue to emerge in wildlife, domestic animals, and humans^47,48^, it is increasingly critical to understand the diverse roles that symbiotic microbes play, including minimizing the harm that pathogens cause their hosts.

## Methods

### Experimental Design

We perturbed the resident ocular microbiome using topical applications of a β-lactam antibiotic (cefazolin, 33 milligrams per milliliter (mg/ml), see Supplementary Material). We selected cefazolin as a broad-spectrum antibiotic^49^ that would target the eubacterial genera that make up the vast majority of the resident bacterial microbiome of house finches^31^, while simultaneously not affecting MG, which is intrinsically resistant to cefazolin due to its lack of a cell wall^50^. We confirmed the sensitivity of members of the finch ocular microbiome to this antibiotic using culture-based methods *in vitro* and *in vivo* (see Supplementary Material). In brief, minimum inhibitory concentrations of cefazolin were measured for all mixed ocular cultures, and they ranged from 0.5–2.5 micrograms per milliliter (µg/ml). *In vivo*, ocular bacterial abundance was significantly reduced post-antibiotic treatment (*p* = 0.027), but did not change for sham controls (*p* = 0.26). We also confirmed, using 16S rRNA gene amplicon sequencing (see Supplementary Material), that the pre-treatment ocular microbiome was dominated by the culturable taxa *Lactococccus* (~76% relative abundance), as we found previously^31^.

We used a full factorial design with three conjunctival antibiotic treatments (no antibiotics, short-term antibiotics, or long-term antibiotics) and two inoculation treatments (MG or sham inoculation), for a total of six treatments with 10 birds/treatment (Table 1). Sex ratios were as close to 1:1 as possible within treatment groups (Table 1). Antibiotics were administered topically three times daily as 15 microliter (µl) drops directly into each eye (see Fig. 1).

Prior to MG inoculation, all birds in the *No Antibiotics* treatment were captured and briefly held, but were otherwise left undisturbed. MG inoculation was done via micropipette droplet directly into both conjunctivae. Birds were inoculated with a total volume of 40 µL of the index MG isolate VA1994 (see Supplementary Material), diluted in Frey’s broth medium with 15% swine serum (FMS) at a total concentration of 2.0 × 10^4^ color changing units (CCU) per ml. Sham birds were inoculated with FMS alone. After MG inoculation, all birds not receiving antibiotic treatment (i.e., *No Antibiotics* and *Short Antibiotics* treatments), regardless of MG inoculation status, were given treatments of sterile saline eye drops during the four-day period that *Long Antibiotics* birds were receiving antibiotics (Fig. 1). This controlled for the possibility that the input of fluid into the conjunctivae three times daily might flush some MG from the conjunctivae.

### Host Species Capture and Housing

Sixty hatch-year house finches were captured June-August 2015 in Montgomery County, VA using cage traps and mistnets (USFWS permit MB158404–0, VDGIF permit 050352). Immediately following capture, all birds were pair-housed at constant day length and temperature and were fed *ad libitum* pelleted diet (Daily Maintenance Diet, Roudybush Inc. Woodland, CA) during a 14-day quarantine (see Supplementary Material). At the end of quarantine, a blood sample was taken from all birds to test for potential exposure to MG prior to capture. Birds that were seronegative for MG using an IDEXX kit enzyme-linked immunosorbent assay (following previously described methods^24^) at 14 days post-capture were considered to be MG-naïve, and only MG-naïve birds were included in MG inoculation groups (Table 1). A subset of birds that were seropositive for MG (n = 20) but MG-negative prior to the start of the experiment (see Supplement) were used in MG control (sham inoculation) experimental groups. All birds were moved to single housing 28 days prior to the start of the study, but all other housing conditions remained unchanged. Cages and sexes were distributed between two rooms, but were randomly assigned to treatment with equal representation of each treatment group and sex within each room. All housing and experimental protocols were approved by and carried out in accordance with guidelines of the Virginia Tech IACUC.

### Experimental sampling

Every 3–4 days post-inoculation (Fig. 1) conjunctival inflammation severity was visually scored and conjunctivae were swabbed to quantify pathogen load via quantitative PCR (qPCR). Inflammation severity of conjunctival tissue was visually scored on a 0 to 3 scale^24^. Briefly, no visible clinical signs was scored as 0, minor swelling around the eye was scored as 1, moderate swelling with occasional conjunctival eversion was scored as 2, and moderate to severe swelling, conjunctival eversion, and noticeable exudate was scored as 3. Inflammation was scored blind to treatment. Scores from each eye were combined within time points to give a composite severity score ranging from 0 to 6 for each individual.

To quantify pathogen load, conjunctivae were swabbed for 5 seconds with a sterile cotton swab dipped in tryptose phosphate broth (TPB). Swabs were swirled in 300 µl of sterile TPB and then wrung out into the sample collection tube. Samples from both eyes were pooled within sampling date for a given individual and frozen at −20 °C until further processing. Genomic DNA was extracted from samples of all MG-inoculated birds (n = 30), and a subset of sham-inoculated birds (n = 18), at six time points just prior to and after inoculation (Fig. 1), with Qiagen DNeasy 96 Blood and Tissue kits (Qiagen, Valencia, CA). Extracted DNA was used to measure overall numbers of MG in the conjunctivae using a qPCR assay targeting the *mgc2* gene of MG using primers and a probe previously described^51^ and qPCR methods previously outlined^52^.

On day 8 post-inoculation, *Mycoplasma* was isolated from both conjunctivae of all MG-inoculated birds via swabbing (Copan FLOQSwabs, Copan Diagnostics Inc., Murrieta, CA) to characterize phenotypic activity of two virulence factors (sialidase activity and cytadherence). Swabs were immediately placed in Remel M5 media (Remel, Waltham, MA) and shipped overnight on cold packs to the University of New England. M5 media was diluted 1:5 in SP-4 media supplemented with 0.5% w/v glucose and incubated for 4 hours (h) at 37 °C. Cultures were monitored for MG growth as determined by acid shift for 3 weeks, and only cultures that showed positive growth were included in the below phenotypic assays (see Supplementary Material).

Sialidase activity was assessed in washed MG cells and quantitated using the fluorogenic substrate 2′-(4-methylumbelliferyl)-α-D-*N*-acetylneuraminic acid (MUAN; Sigma-Aldrich, St. Louis MO) as previously described^53^ (Supplementary Material). Briefly, cell suspensions were prepared for each recovered isolate. Total protein concentration via Bradford assay was determined as a proxy for bacterial cell number in each suspension. MG cell suspensions were incubated for 15 minutes with MUAN, then enzymatic activity was measured by cyan fluorescence at 450 nanometers (nm), excited at 365 nm, with a cutoff filter at 420 nm using a Spectramax M5 platereader. Sialidase activity per mg total protein was calculated and normalized to total protein concentration (U/mg) using a standard curve generated by similar incubation of Type IV *Clostridium perfringens* neuraminidase (Sigma-Aldrich) with MUAN.

To quantify cytadherence, recovered MG isolates were grown to mid-log phase, collected by centrifugation, and quantitated by Bradford assay as above. Briefly, approximately 10^6^ MG cells were fixed with 70% ethanol and stained with the prokaryote-specific fluorescent DNA dye SYTO9. 96-well black polystyrene plates were coated with chicken erythrocyte antigen derived from a 15% chicken erythrocyte suspension (Lampire Biologicals, Pipersville, PA). Stained MG cells were allowed to bind to erythrocyte antigen for 1 h at 37 °C. Unbound cells were then removed by washing with 1x phosphate buffered saline. Bound MG cells were quantified by measuring green fluorescence at 498 nm, excited at 485 nm, with a cutoff filter at 530 nm using a Spectramax M5 platereader. See Supplementary Material for full methods.

### Statistical Analyses

All analyses were conducted in Program R v3.2.1^54^. Pathogen load data were log_10_ transformed prior to analysis.

Because we had repeated measures over time in response to MG inoculation, disease response data (inflammation score and pathogen load) were analyzed using factorial generalized linear mixed effects models (GLMMs) in package {lme4}^55^. Full models included MG inoculation (yes/no), antibiotic treatment (*No*, *Short*, *Long*), post-inoculation day (PID, treated as a factor), and the interactions between MG × antibiotics, MG × PID, and antibiotics × PID as fixed effects. Previous characterization of the house finch ocular microbiome found significant sex differences^31^, so sex was added as a covariate; however, sex was not a significant factor in any model (*p* > 0.46) and was subsequently dropped. Bird ID was included as a random effect to control for repeated individual measurements. Models for both pathogen load and inflammation score were fit using a gaussian distribution with a log-link.

We first examined full models as described above, but because the primary interest was the interaction between MG inoculation and antibiotic treatment, we then limited analyses to MG inoculated birds only (MG+). Both model sets (full and MG+ only) were in agreement, but for simplicity, we report the results of MG+ models here and full models in the supplement (Supplementary Table S2). For MG+ models, antibiotic treatment, PID, and antibiotics × PID were fixed effects, bird ID was included as a random effect, and sex was initially included in all models. Models were simplified using single-term deletion of non-significant terms from Type III Wald chi-square tests (*p* < 0.1 criterion) to arrive at the minimally adequate models.

Because pathogen load and inflammation severity are positively correlated in this system^35^, we also calculated relative inflammation severity (RIS) as a metric of the degree of inflammation controlling for pathogen load. At each time point, inflammation scores were regressed on pathogen loads to calculate individual residuals from each regression and generate a RIS for each bird, which was then analyzed using a gaussian GLMM with RIS as the dependent variable, antibiotic treatment, PID, and antibiotics × PID as predictors, with bird ID as a random effect. Post-hoc Tukey multiple comparisons of means tests were used to determine significant differences among antibiotics treatment groups at each time point in the experiment for all disease response models described above (i.e., inflammation score, relative inflammation severity, and pathogen load models).

We analyzed phenotypic activity of two MG virulence factors, sialidase activity and cytadherence, among treatment groups using a one-way analysis of variance (ANOVA) and post-hoc Tukey multiple comparisons of means tests.

The datasets generated during and/or analyzed during the current study are available from the corresponding author on reasonable request.

## Electronic supplementary material

Supplemental Material
