# Supplementary material for: Resident Microbiome Disruption with Antibiotics Enhances Virulence of a Colonizing Pathogen
Source: Sci Rep. 2017 Nov 23;7:16177. doi: 10.1038/s41598-017-16393-3 (PMC5701009; doi:10.1038/s41598-017-16393-3)
Supplement: Supplementary file 1 — Supplemental Material [file 41598_2017_16393_MOESM1_ESM.pdf]

Supplemental Material

TITLE:

Resident Microbiome Disruption with Antibiotics Enhances Virulence of a Colonizing Pathogen

AUTHORS:

Courtney A. Thomason*, Nathan Mullen, Lisa K. Belden, Meghan May, and Dana M. Hawley

Summary:

Extended experimental and assay methods and details associated with finch quarantine, inoculation, virulence factor assays, and 16S rRNA gene amplicon sequencing are included here. Additionally, *in vivo* and *in vitro* antibiotic sensitivity results and results of full MG × antibiotics models (MG+ and MG- groups) are described.

Post-Capture Quarantine

During quarantine, birds were captured every 3 days to assess potential clinical signs of mycoplasmal conjunctivitis. Any birds with non-zero eye scores were immediately isolated, along with their cage-mates, and were not used in the experiment. During quarantine, all birds were treated with sulfadimethoxine (starting at a high dose 0.496 mg/ml for 5 days, and dropping to low dose 0.26 mg/ml) to control intestinal coccidia infections for a minimum of 60 days post-capture, until the immune system had matured and was capable of controlling coccidia alone. To stop overgrowth of the avian gut yeast *Macrorhabdus ornithogaster*, all birds were given drinking water treated with apple cider vinegar (5mL/L) every other day during the conjunctival

antibiotic treatment period to acidify the gut, as well as probiotic-treated drinking water once a week designed to repopulate the healthy gut flora.

Effects of Antibiotic Perturbation on Development of Mycoplasmal Conjunctivitis

The MG isolate used for experimental MG inoculation, VA1994, was an expansion of the 7th *in vitro* passage of an isolate collected from a house finch with Mycoplasmal conjunctivitis captured in Virginia in 1994 (7994-1-7p, 2/12/09; Ley et al. 1996). Dilutions were calculated from the starting viable count of 2.24×10^7 color changing units (CCU) per milliliter, and inoculum was maintained at -80°C until immediately before dilution.

Each vial of lyophilized cefazolin (NDC 0143-9924-90), the β-lactam antibiotic used in the experiment, was reconstituted with 1X sterile phosphate buffered saline to 330 mg/ml following manufacturer instructions (West-Ward Pharmaceutical Corp., Eatontown, NJ). Stock cefazolin was then diluted into Bausch & Lomb artificial tears to a concentration of 33 mg/ml. Diluted cefazolin was portioned into three aliquots per day to minimize any potential for contamination of antibiotics between treatments and stored at 4°C. This preparation of ophthalmic cefazolin was mixed fresh and discarded every 5 days.

MG Virulence Factor Assays

To assess sialidase enzyme activity, cell suspensions were prepared for each recovered MG isolate by collecting the bacteria by centrifugation, washing the pellets three times with 1x phosphate buffered saline (PBS), and resuspending the final cell pellet in 100 µL PBS. Ten µL was removed to determine the total protein concentration of each suspension as a proxy for

bacterial cell number by Bradford assay, according to the manufacturer's instructions (Thermo Fisher Scientific). MG cell suspensions were then incubated for fifteen minutes with MUAN. Enzymatic activity was measured by cyan fluorescence at 450 nm, excited at 365 nm with a cutoff filter at 420 nm using a Spectramax M5 platereader. The amount of sialidase activity per mg total protein was calculated from a standard curve generated by similar incubation of Type VI *Clostridium perfringens* neuraminidase (Sigma-Aldrich) with MUAN. Enzymatic unit readings for each MG isolate were then normalized to the total protein concentration, generating sialidase measurements reported in U/mg protein.

To quantify cytadherence, recovered MG isolates were grown to mid-log phase, collected by centrifugation, and quantitated by Bradford assay, as described above. 96-well black polystyrene plates were coated with 15% chicken erythrocyte suspension (Lampire Biologicals, Pipersville, PA) in bicarbonate buffer (pH = 9.4). Following osmotic lysis, unbound erythrocytes and liberated hemoglobin were removed by washing 3 times with 1x PBS. Coated wells were then blocked with 5% bovine serum albumin. Approximately 10^6 MG cells were fixed with 70% ethanol and stained with the prokaryote-specific fluorescent DNA dye SYTO9 according to the manufacturer's instructions (Molecular Probes/Thermo Fisher Scientific). Stained MG cells were allowed to bind to erythrocyte antigen for 1 hour at 37° C, after which unbound cells were removed by washing with 1x PBS. Bound MG cells were quantified by measuring green fluorescence at 498 nm excited at 485 nm with a cutoff filter at 530 nm using a Spectramax M5 platereader.

Statistical Analysis

Because our qPCR assay is highly sensitive and subject to low levels of background contamination (Leon et al, in review), we applied a conservative threshold of infection (1349 copies of pathogen; Adelman et al, 2015) to ensure results were not skewed by contamination. One bird from the MG-inoculated, *Long Antibiotics* treatment was omitted from all analyses because it was found to have pathogen load above this conservative threshold of infection one day prior to MG inoculation, and thus we could not be confident that this bird was truly naïve to MG at the time of inoculation.

Although some finches in the MG sham experimental groups (which never received MG in our experiments) were seropositive, indicating potential prior exposure to MG in the wild, all finches in these experimental groups were MG-negative via qPCR at the start of the experiment. Two individuals in these groups had pathogen loads well below the conservative threshold described above and in Leon and Hawley (2017), consistent with background contamination of our qPCR assay. Thus, birds in our MG sham groups did not have active *M. gallisepticum* infection despite some individuals harboring antibodies suggestive of prior exposure.

16S rRNA gene amplicon sequencing

Genomic DNA from select time-points that was used for qPCR of MG (pre-inoculation day -1 and post-inoculation day 8) was also used for 16S rRNA gene amplicon sequencing of the V4 region using primers 515F and barcoded 806R (Caporaso et al, 2012), and following methods outlined in Thomason et al (2017). However, overall these samples had low DNA quantity (2.13 – 17.7 ng/uL DNA concentrations) relative to other samples on the same plate, with samples from antibiotic-treated birds being of particularly low quality. Doan et al. (2106) similarly found that human conjunctival microbiomes are paucibacterial compared to buccal mucosa and facial

skin microbiomes, with quantitative PCR indicating the presence of less than 40 bacterial cells per conjunctival swab. As a result of poor sequencing data, we did not complete a full analysis of these samples. We produced an OTU table that was rarefied at 1100 reads, and only used a subset of samples (*No Antibiotics* sham-inoculated controls on PID -1 only) to confirm that the dominant bacterial ocular community member was *Lactococcus,* as was also the case in our prior work (Thomason et al, 2017). *Lactococcus* in the present study represented 76% ± 0.02 of the relative abundance of the resident ocular microbiome prior to MG inoculation, which was very similar to our previous findings (Thomason et al, 2017).

Sensitivity of the Resident Community to Antibiotic Perturbation

Our prior work (Thomason et al, 2017) indicates that the dominant members of the house finch community are culturable, with *Lactococcus spp*. making up the majority of the relative abundance. Thus, we confirmed that the ocular bacterial community responded to our antibiotic perturbation using two culture-based methods. First, we examined effects of antibiotic perturbation on the culture-permissive component of the house finch conjunctiva *in vivo* using a separate set of birds, to minimize effects of depletion of the bacterial microbiome in response to repeated sampling. Because the antibiotic used can act in a bacteriostatic manner, culture-based methods allowed us to detect functional differences in the viability of the conjunctival bacterial community following antibiotic treatment. We then used *in vitro* plating methods to confirm the sensitivity of mixed ocular cultures and pure cultures of *Lactococcus* spp. isolated from house finch conjunctiva to the antibiotic, cefazolin.

In Vivo Antibiotic Sensitivity Experiment

To examine the sensitivity of the cultivatable ocular community members to the antibiotic we used, ocular samples were collected prior to and following antibiotic treatment (Figure 1; M* samples) for 17 total birds (*Short Antibiotics*: n=10; *No Antibiotics Control*: n=7), 10 of which received topical antibiotic treatment as described in the Main Text, but in the absence of MG inoculation. Seventeen hatch-year house finches (7 males, 10 females) were captured between June and August 2016 in Montgomery County, VA using cage traps (under federal and state permits USFWS #MB158404-0 and VDGIF #056090) and housed under identical conditions as those described above. Notable differences in the quarantine period were treatment with 2 rounds of sulfadimethoxine treatment (0.469 mg/ml for 6 days) immediately upon capture and again 2 weeks later. All birds were bled and screened for MG exposure via ELISA using an IDEXX MG Ab Test kit (IDEXX, Westbrook, Maine). All birds were single housed seven days prior to the beginning of antibiotic treatment.

The same preparation of cefazolin β-lactam antibiotic was used following the same treatment schedule (*Short Antibiotics* and *No Antibiotics* control) as used in the primary experiment (see Main Text).

To culture the resident ocular microbiome, conjunctivae were swabbed for 10 seconds with flocked swabs (Copan FLOQSwabs, Copan Diagnostics Inc., Murrieta, CA) pre-dipped in sterile saline. Both conjunctival swabs from a given bird were immediately combined in 3 mL brain heart infusion (BHI) medium (Thermo Fisher Scientific), vortexed, and incubated for 24 hours at 37°C prior to overnight transport on cold packs to the University of New England. Upon arrival,

the optical density (OD) of each sample was measured (λ = 600 nm) to quantify overall bacterial growth among samples, our metric for quantification of the culture-permissive members of the ocular bacterial community.

Cultures were then streaked to obtain isolated colonies on both BHI agar (Thermo Fisher Scientific/Remel) and MRS agar (Teknova, Hollister, CA) to allow for inclusive growth and to select for lactic acid bacteria, respectively. Colonies recovered on MRS agar were positively identified as *Lactococcus* spp. by amplification and dideoxy sequencing of the 16S ribosomal RNA gene (Decker et al, 2013) or biochemical profiling using the BBL Crystal ID system for Gram positive organisms (Becton, Dickinson, and Company [BD], Franklin Lakes, NJ). Colonies isolated on BHI agar were divided into "morphotypes" but not positively identified. Recovered isolates were preserved in 10% dimethylsulfoxide at -80°C for subsequent studies.

Culturable microbiome OD data were analyzed using a one-way ANOVA to compare OD values of treatment groups within sampling time points, and a paired t-test comparing OD values for swabs collected from birds before and after perturbation within the *No Antibiotics* control and *Short Antibiotics* treatment groups.

In Vivo Experiment - Results

Our topical antibiotic perturbation significantly reduced the abundance of culture-permissive resident bacteria, measured via the OD of culture swab growth media (post-treatment: F=6.00, df=1, p=0.027; Fig. S1). Although culture success appeared to decrease between sampling events, control birds that did not receive antibiotics showed no statistically significant change in

bacterial abundance (paired t-test: t = 1.24, df = 6, p = 0.26). There were also no differences in culture success amongst the two groups of birds prior to antibiotic perturbation (pre-treatment: F=0.01, df=1, p=0.91), suggesting that antibiotic treatment effectively disrupted the resident bacterial ocular microbiome.

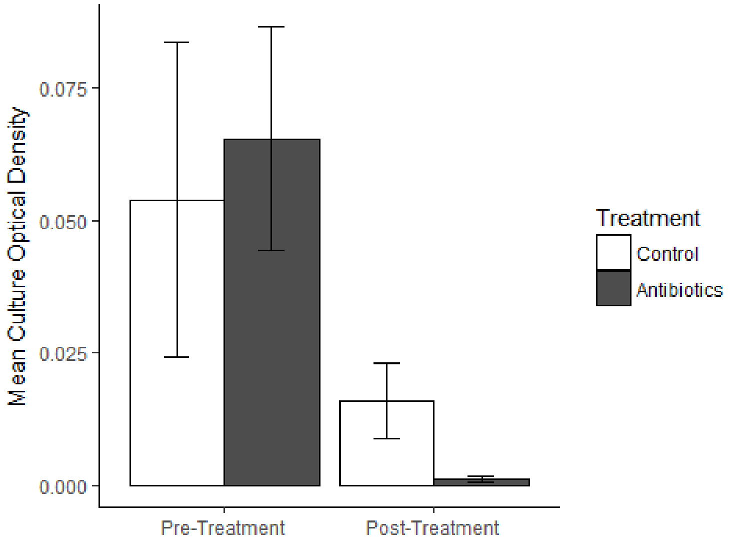

Figure S1. Topical antibiotic treatment results in significantly lower culturing success of culture-permissible bacteria, quantified with culture broth optical density, in the resident ocular microbiome.

In vitro Antibiotic Sensitivity of Mixed Ocular Isolates and Lactococcus Isolates

Antibiotic susceptibility testing (AST) for cefazolin was performed on mixed cultures from birds in the *in vivo* sensitivity study (methods and result above). We used serial, two-fold broth microdilution using cation-adjusted Mueller-Hinton broth (CAMHB, Difco, BD Sparks MD). AST broth tubes were incubated at 37˚C for 24 h in ambient air. Minimum inhibitory concentrations of cefazolin were measured for all mixed cultures, and they ranged from 0.5-2.5 ug/mL, all within the “susceptible” range for cefazolin.

Because *Lactococcus* spp. make up the vast majority (~76%) of relative abundance of the resident ocular microbiome of birds in our study, we also performed *in vitro* antibiotic susceptibility testing (as described above) for all colonies identified as *Lactococcus* spp. (see above). *Lactococcus* isolates were subcultured from mixed broth cultures (1 per bird; obtained from *in vivo* experiment) onto BHI agar plates, and colonies were picked and suspended in saline to achieve a concentration equivalent to a 0.5 McFarland standard inoculum (CLSI M07-A10; January, 2015). The minimum inhibitory concentration for *Lactoccocus* spp. isolates was 0.5 µg/mL of cefazolin, indicating that *Lactoccocus* isolated from the conjunctivae of house finches is strongly sensitive to the antibiotic used.

Effects of Cefazolin on Sialidase Activity

To ensure that residual cefazolin did not influence the sialidase activity of *M. gallisepticum* isolates, we performed replicate sialidase assays (using methods described in the main text) with and without the addition of cefazolin (0.5 mg/mL in saline; 50µg per assay x 3 replicates / each). We found no effect of cefazolin on sialidase activity of *M. gallisepticum* (cefazolin added (n=3): mean±s.d. 0.0795±0.0020; No cefazolin (n=3): mean±s.d. 0.0792±0.0011; t=0.28; p=0.79).

Supplemental Results from Primary Experiment (Main Text)

Here, we report the full models, including house finches that received sham inoculations of media only, that mirror the models reported in the main text.

Antibiotic Treatment and Pathogen Load

As expected, pathogen loads were significantly higher in birds inoculated with MG rather than media alone on all time points except the pre-inoculation time point (MG × Post-inoculation Day (PID): $X^2 = 35.3$, df = 5, $p < 0.001$; post-hoc Tukey contrasts, least-squares means: $p < 0.04$ from PID 5 to PID 26). The effect of antibiotics on pathogen load also varied with time post-inoculation (antibiotics × PID: $X^2 = 45.36$, df = 10, $p < 0.001$). Antibiotic-disruption resulted in significantly higher pathogen loads in house finches that received antibiotic treatment at PID 5 (post-hoc Tukey contrasts, least-squares means: control versus short, p = 0.28; control versus long, p = 0.006). There were no effects of house finch sex alone ($X^2 = 0.04$, df = 1, p = 0.84), or in combination with antibiotic disruption ($X^2 = 0.49$, df = 2, p = 0.78), on pathogen loads. There were no significant differences in pathogen load between short and long antibiotics treatment groups at any time point (post-hoc Tukey contrasts, least-squares means: short versus long, p > 0.25).

Antibiotic Treatment and Development of Pathology

Inflammation severity was significantly more severe in house finches that were inoculated with MG versus media alone, and the strength of this effect changed over the course of infection (MG × PID: $X^2 = 167.9$, df = 7, $p < 0.001$). Differences in inflammation were highest between PIDs 8-19 (post-hoc Tukey contrasts, least-squares means: sham versus MG-inoculated, $p < 0.007$). Antibiotic treatment resulted in significantly increased inflammation severity compared to *No Antibiotics* control finches, and this effect also varied with time post-inoculation (antibiotics × PID: $X^2 = 38.3$, df = 14, $p < 0.001$). Finches that received antibiotics, regardless of short or long treatment, had higher composite eye scores on PIDs 12 and 15 (post-hoc Tukey contrasts, least-

squares means: PID 12 control versus short, $p = 0.03$; PID12 control versus long, $p = 0.07$; PID 15 control versus short, $p = 0.002$; PID15 control versus long, $p = 0.09$; short versus long, $p > 0.08$ at all time points), compared to *No Antibiotics* control finches. House finch sex alone (sex: $X^2 = 0.31$, df = 1, $p = 0.57$) or in combination with antibiotic treatment (sex × antibiotics: $X^2 = 0.61$, df = 2, $p = 0.73$) did not significantly impact development of tissue inflammation.

Antibiotic Treatment and MG Cytadherence

Just as with sialidase activity, MG cytadherence increased significantly after treatment with cefazolin, regardless of length of antibiotic treatment ($X^2 = 12.4$, df = 2, $p < 0.001$). This change in cytadherence was tightly correlated with the increase in sialidase activity (R=0.98).

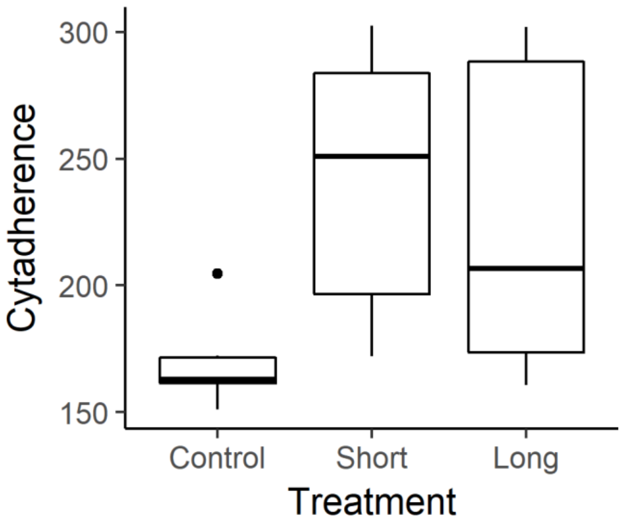

Effects of prior β-lactam antibiotic perturbation on Mycoplasma gallisepticum *cytadherence.*

Table S1. Post hoc comparisons for MG+ inflammation score, relative inflammation, and pathogen load models on each day post-inoculation (PID). Reported values are Tukey LSMeans ± SE with p-values corrected for multiple comparisons in parentheses. All estimates are in reference to the first treatment group listed in each pairwise comparison. Bolded values indicate statistically significant differences.

Response Variable	PID	*No Antibiotics - Short Antibiotics*	*No Antibiotics - Long Antibiotics*	*Short Antibiotics - Long Antibiotics*
Inflammation Score	-1	0.17 ± 0.28 (0.810)	0.11 ± 0.28 (0.921)	-0.06 ± 0.29 (0.974)
	2	0.16 ± 0.28 (0.834)	0.09 ± 0.28 (0.938)	-0.06 ± 0.29 (0.973)
	5	0.16 ± 0.28 (0.834)	-0.08 ± 0.27 (0.954)	-0.24 ± 0.27 (0.657)
	8	-0.45 ± 0.23 (0.115)	-0.27 ± 0.23 (0.483)	0.18 ± 0.21 (0.668)
	12	**-0.46 ± 0.19 (0.047)**	-0.36 ± 0.19 (0.147)	0.09 ± 0.18 (0.868)
	15	**-0.56 ± 0.19 (0.009)**	-0.35 ± 0.19 (0.160)	0.21 ± 0.18 (0.484)
	26	-0.31 ± 0.22 (0.330)	0.12 ± 0.24 (0.865)	0.44 ± 0.23 (0.129)
Relative Inflammation Score	-1	0.01 ± 0.31 (0.999)	-0.00 ± 0.32 (0.999)	-0.01 ± 0.32 (0.999)
	5	0.06 ± 0.31 (0.981)	-0.10 ± 0.32 (0.948)	-0.16 ± 0.32 (0.875)
	8	-0.63 ± 0.31 (0.109)	-0.38 ± 0.32 (0.467)	0.26 ± 0.32 (0.703)
	12	**-0.82 ± 0.31 (0.027)**	**-1.08 ± 0.32 (0.003)**	-0.26 ± 0.32 (0.697)
	15	-0.52 ± 0.31 (0.224)	-0.70 ± 0.32 (0.078)	-0.18 ± 0.32 (0.835)
	26	-0.26 ± 0.31 (0.684)	0.20 ± 0.32 (0.804)	0.46 ± 0.32 (0.322)
Pathogen Load	-1	0.04 ± 0.42 (0.994)	0.39 ± 0.52 (0.730)	0.35 ± 0.53 (0.788)
	5	-0.49 ± 0.24 (0.096)	**-0.61 ± 0.24 (0.028)**	-0.12 ± 0.22 (0.847)
	8	-0.10 ± 0.21 (0.882)	-0.06 ± 0.21 (0.952)	0.04 ± 0.21 (0.983)
	12	-0.14 ± 0.21 (0.774)	0.19 ± 0.22 (0.650)	0.33 ± 0.21 (0.261)
	15	-0.37 ± 0.22 (0.211)	-0.05 ± 0.23 (0.973)	0.32 ± 0.22 (0.320)
	26	-0.52 ± 0.29 (0.168)	-0.30 ± 0.30 (0.572)	0.22 ± 0.26 (0.678)

Table S2. Results of full host and pathogen disease response models analyzing the effects of antibiotic ocular microbiome disruption on development of Mycoplasmal conjunctivitis in house finches. Bold values indicate statistical significance following Type III Wald Chi-square tests.

Response	*Predictor*	X^2	*df*	*p-value*
Log_{10} MGC2 pathogen load	sex	0.04	1	0.84
	MG × antibiotics	0.94	2	0.62
	**MG × PID**	**35.31**	**5**	**< 0.001**
	**antibiotics × PID**	**45.36**	**10**	**< 0.001**
	antibiotics × sex	0.49	2	0.78
Inflammation Severity Score	sex	0.31	1	0.57
	MG × antibiotics	1.92	2	0.38
	**MG × PID**	**167.92**	**7**	**< 0.001**
	**antibiotics × PID**	**38.31**	**14**	**< 0.001**
	antibiotics × sex	0.61	2	0.73

Literature Cited

Adelman JS, Moyers SC, Farine DR, Hawley DM. (2015). Feeder use predicts both acquisition and transmission of a contagious pathogen in a North American songbird. Proc R Soc Lond B 282:20151429.

Caporaso JG, Lauber CL, Walters WA, Berg-Lyons D, Huntley J, Fierer N, et al. (2012). Ultra-high-throughput microbial community analysis on the Illumina HiSeq and MiSeq platforms. ISME J 6:1621-1624. doi: 10.1038/ismej.2012.8.

Decker C, Olu K, Arnaud-Haond S, Duperron S. (2013). Physical proximity may promote lateral acquisition of bacterial symbionts in vesicomyid clams. PloS One 8:e64830.

Doan T, Akileswaran L, Andersen D, Johnson B, Ko N, Shrestha A, et al. (2016). Paucibacterial microbiome and resident DNA virome of the healthy conjunctiva. Invest Ophthalmol Vis Sci 57:5116-5126.

Hawley DM, Grodio J, Frasca S Jr, Kirkpatrick L, Ley DH. (2011). Experimental infection of domestic canaries (*Serinus canaria domestica*) with *Mycoplasma gallisepticum*: a new model system for a wildlife disease. Avian Pathol 40:321-327.

Leon, AE and Hawley, DM. (2017). Host responses to pathogen priming in a natural songbird host. EcoHealth doi: 10.1007/s10393-017-1261-x.

Ley, D.H., Berkhoff, J.E., and McLaren, J.M. (1996). Mycoplasma gallisepticum isolated from house finches (Carpodacus mexicanus) with conjunctivitis. Avian Dis. 40: 480-483.

Pflaum, K., Tulman, E.R., Beaudet, J., Liao, X., & Geary, S.J. (2016). Global changes in *Mycoplasma gallisepticum* phase-variable lipoprotein gene vlhA expression during *in vivo* infection of the natural chicken host. Infect. Immun. **84**, 351-355.

Thomason CA, Leon A, Kirkpatrick LK, Belden LK, Hawley DM. (2017). Eye of the Finch: Characterization of the Ocular Microbiome of House Finches in Relation to Mycoplasmal Conjunctivitis. Environ Microbiol 19:1439-1449.
